# The prevalence of multiple drug resistant urinary tract infections: A single-centered, observational retrospective study in King Abdulaziz Specialized Hospital, Taif, Saudi Arabia

**DOI:** 10.15537/smj.2022.43.8.20220238

**Published:** 2022-08

**Authors:** Faisal K. Alhomayani, Naif M. Alazwari, Mohammed S. Alshhrani, Ali S. Alkhudaydi, Abdullah S. Basaba, Tariq M. Alharthi, Muhanad M. Alghamdi, Abdulaziz S. Aljuaid, Nasser M. Alosimi, Abdulmajeed M. Alqethami

**Affiliations:** *From the Department of Nephrology and Kidney Transplant (Alhomayani); from the Department of Internal Medicine (Aljuaid, Alqethami), College of Medicine, Taif University; and from the Department of Internal Medicine (Alazwari, Alshhrani, Alkhudaydi, Basaba, Alharthi, Alghamdi, Alosimi), King Abdulaziz Specialist Hospital, Taif, Kingdom of Saudi Arabia*.

**Keywords:** prevalence, multiple drug resistance, UTI

## Abstract

**Objectives:**

To determine the prevalence and patterns of antibiotic resistance, risk factors, and appropriate empiric therapy for multidrug-resistant *Enterococcus* (MDRE) urinary tract infections (UTIs) at King Abdulaziz Specialist Hospital (KAASH), Taif, Saudi Arabia.

**Methods:**

All patients attending KAASH with MDRE UTIs between January 2018 and December 2020 were enrolled in the study. After ethical approval, data were analyzed.

**Results:**

The most common causative organisms were Gram-negative and anaerobes, and the most sensitive antibiotics were ciprofloxacin and ceftriaxone.

**Conclusion:**

Based on our analyses, regular culture and sensitivity should be made routine to gather information regarding susceptibility patterns, thereby reducing drug resistance in our setups.

**M**ultidrug resistance (MDR) urinary tract infection (UTI) defines as non-susceptibility to at least one antimicrobial in 3 or more classes. The criteria to diagnose UTI is symptoms of dysuria, frequency or urgency associated with: i) urinalysis: pyuria + bacteriuria ± hematuria ± nitrites; ii) urine culture (clean-catch midstream or straight-cath), and iii) if: ≥105 colony forming unit (CFU)/mL in women, ≥103 CFU/mL in men.

The incidence of MDR or in pathogenic and opportunistic bacteria has been increasing in the recent years. These MDR bacteria have also created immense clinical problems in cancer and immune-compromised patients. The most important MDR bacteria on the global scale include gram-positive (methicillin-resistant *Staphylococcus aureus*, vancomycin-resistant *Enterococci*), gram-negative bacteria (members of *Enterobacteriaceae* producing plasmid-mediated extended-spectrum β-lactamase), and others like *Pseudomonas aeruginosa* and *Mycobacterium tuberculosis*.^1^ Careless and injudicious use of antibiotics and empirical antimicrobial therapy have been the major contributing factors in the emergence of MDR bacteria. Antibiotic resistance is an emerging global problem.

Urinary tract infection is a common bacterial disease, often contributing to frequent morbidity in out-patients and hospitalized patients. Clinical experience has indicated the presence of numerous cases of antibiotic resistance to common antibiotics by uropathogens in both developed and developing countries. Resistances to newer and more potent antimicrobials are no exceptions, making therapeutic options very limited to certain antimicrobial agents, such as carbapenem, colistin, and fosfomycin.^2,3^

Antimicrobial resistance (AMR) is an important health and economic burden, and raises the threat of a post-antibiotic future. Antimicrobial resistance also negatively affects patient health status and lengthens the stay of hospital.^4,5^ Updated knowledge of causal bacteria and their susceptibility patterns are important for proper selection and use of antibiotics as well as for an appropriate prescribing policy.

This study aimed to assess the prevalence of MDR UTI and determine drug-resistant urinary pathogens and appropriate empiric therapy for MDRE UTIs in King Abdulaziz Specialist Hospital (KAASH), Taif, Saudi Arabia. This knowledge could help formulate and monitor the antibiotic policy and proper empirical therapy.^2,6^

## Methods

This is a single-centered, observational retrospective study carried out in KAASH, Taif, Saudi Arabia. After reviewing 321 UTI patients at KAASH, 240 patients with positive urine cultures attending KAASH with MDRE UTIs between January 2018 and December 2020 were included in this study. Patients less than 18 years old and patients who had asymptomatic bacteriuria were excluded from the study.

Approval from the Ethical Committee and hospital administrative authorities was obtained. Patients who had UTIs were identified via KAASH electronic patient record (total of 321 patients). In addition, the patient’s medical records were searched for any missing information. Due to the study’s retrospective nature, informed consent was not needed. In the study, data were collected from patients’ medical records found in the database. The confidentiality was maintained by converting the patient’s medical records into serial numbers after extraction in a secured computer at KAASH throughout the study period. Also, to maintain the confidentiality of the data, the only people who have the right to access the data are the supervisor and the 6 research group members.

The data collection form was completed with the following information regarding each included patient: demographics, pregnancy status, underlying medical comorbidities, relevant surgical history including urological procedure and obstructive uropathy, prior UTI, prior antibiotic use within 3 months, UTI diagnoses, empiric treatments, causative bacteria, and antibiotic susceptibility. A total of 240 patients had positive culture while 81 had a negative culture. The gathered data were reviewed and analyzed.

### Statistical analysis

Data were extracted from the data collection form, and the Statistical Package for the Social Sciences, version 26.0 (IBM Corp., Armonk, NY, USA) was used for all statistical analyses. Categorical variables were expressed as percentages. Numerical data was shown by histogram to assess the distribution. If normally distributed, means and standard deviation were used; if skewed, median and interquartile were used.

## Results

Table 1 shows that approximately 47.1% were over 70 years old, followed by 29.9% aged 50-70 years. More than half (55.8%) were female. Almost all patients had a chronic medical disease, such as diabetes mellitus (DM)(62.2%), hypertension (57.7%), and bedridden (36%) Table 1 shows that nearly half (46.2%) of the patients had a MDR positive UTI culture.

**Table 1:** - Frequency distribution of the biosociodemogaphic characteristics of the studied patients.

Characteristics	n (%)
***Age, mean±SD (range)***	
<30	18±7.5
30-50	38±15.8
50-70	71±29.6
>70	113±47.1
***Gender***	
Female	134 (55.8)
Male	106 (44.2)
***Positive culture UTI***	
MDR	111 (46.3)
Non MDR	129 (53.8)
***Chronic medical disease (MDR)***	
Hypertension	64 (57.7)
Diabetes mellitus	69 (62.2)
Congestive heart failure	13 (11.7)
Chronic lung disease	3 (2.7)
Chronic liver disease	2 (1.8)
Chronic renal failure	31 (27.9)
Bedridden	40 (36.0)
Ventral nervous system disorders like CVA	28 (25.2)
Malignancy	7 (6.3)
Human immunodeficiency virus	0 (0.0)
***Risk factors (MDR)***	
Previous hospitalization (≤1 year)	66 (59.5)
UTI in (≤1 year)	48 (43.2)
Previous antibiotics (≤3 months)	40 (36.0)
Urinary catheters (≤3 months)	57 (51.3)
Urologic diseases	34 (30.6)
Immunosuppressive/chemotherapy drugs (≤3months)	12 (10.8)

Values are presented as number and percentage (%). SD: standard deviation. UTI: urinary tract infection, MDR: multidrug resistance, CVA: cerebral vascular attack

Among all UTI patients, 74.8% 0had a positive culture, while 25.2% had none, and among all positive UTI patients, 129 (53.8%) of them were non-MDR, while 111 (46.3%) were representing MDR.

Table 2 shows that among MDR group, cipro (20.7%), meropinum or meropenem (24.3%), tazocine (15.3%), augmentin (7.3%), levofloxacin (4.5%), and linezolid (3.6%) were the most common antibiotics of bacterial resistance.

**Table 2:** - Distribution of drug susceptibility in *multidrug resistance* group (N=111).

Variables	n (%)
Cipro	23 (20.7)
Pipracillin tazbactam	1 (0.9)
Ceftrixone	9 (8.1)
Ceftazidime	1 (0.9)
Meropinum or meropenem	27 (24.3)
Levofloxacin	5 (4.5)
Metronedazole	1 (0.9)
Tazocine	17 (15.3)
Augmentin	8 (7.2)
Linezolid	4 (3.6)
Levofluxacin	1 (0.9)
Vancomycin	1 (0.9)
Cefepime	1 (0.9)
Doxy	1 (0.9)
Tigecycline	1 (0.9)
Cefazolin	1 (0.9)

Values are presented as number and percentage (%).

Table 3 shows significant differences between the 2 groups concerning age (*p*<0.001), gender (*p*=0.038), presence of UTI in ≤1 year (*p*<0.001), and urologic diseases (*p*=0.008) with increased incidence of MRD among those older than 70 years, male patients, and those having UTI in ≤1 year or urologic diseases.

**Table 3:** - Correlation between age, gender, *urinary tract infection* in ≤1 year, urologic diseases, and presence of multiple drug resistance.

Variables	Groups			Chi-square	
	MDR	Non-MDR	Total	X^2^	*P*-value
***Age***					
<30	3 (2.7)	15 (11.6)	18 (7.5)	20.061	<0.001^*^
30-50	13 (11.7)	25 (19.4)	38 (15.8)		
50-70	27 (24.3)	44 (34.1)	71 (29.6)		
>70	68 (61.3)	45 (34.9)	113 (47.1)		
***Gender***					
Female	54 (48.6)	80 (62.0)	134 (55.8)	4.323	0.038^*^
Male	57 (51.4)	49 (38.0)	106 (44.2)		
***UTI in ≤1 year***					
No	1 (56.8)	127 (98.4)	190 (79.2)	62.88	<0.001^*^
Yes	48 (43.2)	2 (1.6)	50 (20.8)		
***Urologic diseases***					
No	78 (70.3)	109 (84.5)	187 (77.9)	7.01	0.008^*^
Yes	33 (29.7)	20 (15.5)	53 (22.1)		

Values are presented as number and percentage (%).

* Significant at *p*<0.05. MDR: multiple drug resistance, UTI: urinary tract infection

Table 4 clarifies that organisms (60.8%) other than *E. coli* (17.0%, 17.8%), *Klebsiella spp* (18.3%, 20.16%), and *Proteus spp* (12.5%, 18.6%) were the most common cause among MDR, non-MDR patients, and cultures of non-MDR organisms. Anaerobes (85.6%) and gram-negative (82.9%) were the most common MDR organisms. Ciprofloxacin (36.9%) and ceftriaxone (26.1%) were the empirical antibiotics for culture-positive MDR.

**Table 4:** - Frequency distribution of patients’ culture profile.

Variables	n (%)
***Common organisms (MDR and non-MDR)***	
*E. coli*	41 (17.1)
*Klebsiella spp*	44 (18.3)
*Proteus spp*	30 (12.5)
*Enterococcus spp*	21 (8.8)
Others	146 (60.8)
***Non-MDR organisms***	
*E. coli*	23 (17.8)
*Klebsiella spp*	26 (20.2)
*Proteus spp*	24 (18.6)
*Enterococcus spp*	14 (10.9)
Mixed MDR	5 (3.9)
Others	24 (18.6)
***MDR organisms***	
Gram-positive	19 (17.1)
Gram-negative	92 (82.9)
Anaerobes	95 (85.6)
Aerobes	16 (14.4)
***Empirical antibiotics for culture positive MDR***	
Ciprofloxacin	41 (36.9)
Amoxicillin/clavulanic acid	15 (13.5)
Piperacillin/tazobactam	11 (9.9)
Ceftriaxone	29 (26.1)
Ceftazidime	9 (8.1)
Others	6 (5.4)

Values are presented as number and percentage (%). MDR: multidrug resistant, *E. coli: Escherichia coli*, spp: several species

The resistant strains of uropathogens (against tested antibiotics) are shown in Table 5. There was significant resistance of organisms against ampicillin (*p*=0.004), amoxicillin/clavulanic acid (*p*=0.005), ceftriaxone (*p*<0.001), tetracycline (*p*=0.002), and co-trimoxazole (*p*<0.001). Ampicillin, ceftriaxone, and amoxicillin/clavulanic acid were effective antibiotics for *Klebsiella spp*, tetracycline was effective against *Enterococcus spp*, and co-trimoxazole was effective for *E. coli*.

**Table 5:** - Frequency of antimicrobial resistance of common multiple drug resistance uropathogens (N=87).

Antibiotics	MDR organisms				Chi-square	
	*E.coli* (n=23)	*Klebsiella spp* (n=26)	*Proteus spp* (n=24)	*Enterococcus spp* (n=14)	X^2^	*P*-value
Ciprofloxacin	19 (82.6)	24 (92.3)	22 (91.7)	14 (100)	3.360	0.339
Gentamicin	13 (56.5)	16 (61.5)	18 (75.0)	7 (50.0)	2.874	0.411
Ampicillin	9 (39.1)	20 (76.9)	12 (50.0)	3 (21.4)	13.187	0.004^*^
Amoxcillin/clavulanic acid	10 (43.5)	21 (80.8)	15 (62.5)	4 (28.6)	12.649	0.005^*^
Piperacillin/tazobactam	3 (13.0)	3 (11.5)	1 (4.2)	0 (0.0)	2.918	0.404
Ceftriaxone	9 (39.1)	22 (84.6)	11 (45.8)	2 (14.3)	20.849	<0.001^*^
Ceftazidime	7 (30.4)	11 (42.3)	9 (37.5)	1 (7.1)	5.586	0.134
Cefepime	7 (30.4)	12 (46.2)	10 (41.7)	1 (7.1)	6.915	0.075
Tetracycline	1 (4.4)	0 (0.0)	2 (8.3)	5 (35.7)	15.093	0.002^*^
Co-trimoxazole	15 (65.2)	6 (23.1)	9 (37.5)	1 (7.1)	15.555	<0.001^*^
X^2^	46.953	99.301	63.349	56.925		
*P*-value	<0.001^*^	<0.001^*^	<0.001^*^	<0.001^*^		

^*^ Significant relationship. Values are presented as number and percentage (%). MDR: multiple drug resistance, *E. coli: Escherichia coli*, spp: several species

Table 6 shows the multivariate logistic regression analysis of risk factor of MDR UTI patients. It was found that having UTI in ≤1 year (having an infection with *Proteus mirabilis*) and being on ceftrixone treatment were independent predictors (risk factors) for MDR among the studied UTI patients.

**Table 6:** - Multivariate logistic regression analysis of risk factor of multiple drug resistance in *urinary tract infection* patients.

Variables	B	S.E.	Wald	*P*-value	Odd ratio	95% CI	
						Lower	Upper
Age	0.01	0.009	3	0.083	0.98	0.98	1
Gender	0.57	0.35	2.65	0.1	0.58	0.28	1.12
UTI in ≤1 year	3.4	0.77	19.25	<0.001^*^	0.03	0.007	0.15
Urologic diseases	0.51	0.4	1.58	0.209	0.59	0.26	1.33
*Escherichia coli*	0.001	0.41	0.001	0.999	0.99	0.44	2.24
*Klebsiella pneumoniae*	0.46	0.44	1.1	0.292	0.62	0.26	1.49
*Proteus mirabilis*	1.5	0.54	7.67	0.006^*^	0.22	0.07	0.64
*Enterococcus faecium*	1.08	0.72	2.22	0.136	0.33	0.08	1.4
Cipro	0.25	0.43	0.34	0.556	1.29	0.54	3.05
Ceftrixone	1.83	0.69	3.94	0.047^*^	0.25	0.06	0.98
Meropinum or meropenem	0.45	0.63	0.5	0.477	0.63	0.18	2.21
levofloxacin	0.02	0.89	0.001	0.978	0.97	0.17	5.58
Tazocine	0.56	0.47	1.4	0.236	0.56	0.22	1.44
Augmentin	1.02	0.74	1.19	0.166	0.35	0.08	1.53
Linezolid	0.55	1.6	0.11	0.731	0.57	0.02	13.26
Constant	3.09	0.8	14.94	<0.001	22.15		

^*^ Significant relationship.UTI: urinary tract infection, CI: confidence interval, Exp (B): expected beta, S.E: standard error

## Discussion

Urinary tract infections are the second most common type of infections in human medicine in the United States and Europe, and the third most common (following respiratory tract infections and gastrointestinal infections) infectious pathologies worldwide, representing an important factor of morbidity and mortality, both among out-patients and hospitalized patients (in the latter group, they may represent 25-50% of infections overall).^7^ Urinary tract infections are a considerable economic burden for healthcare institutions and national economies; additionally, they have a substantial economic impact resulting in lost working days. It often requires suitable effective antibiotics based on the correct identification of the causative organisms.^8,9^

Our study revealed that positive UTI cultures were more common among female patients over 50 years of age. Most patients had hypertension DM as a chronic disease. They were previously hospitalized and had previous UTI and catheterization. The most common organisms among non-MDR were *Klebsiella spp, Proteus spp*, and *E. coli*, while the most common among the MDR patients were gram-negative and anaerobes. The most sensitive antibiotics were ciprofloxacin and ceftriaxone.

These results were in line with a study by Kazmi et al,^10^ who confirmed that this infection was suffered mostly by females in Saudi Arabia. They also added that almost 50% of adult women have an episode of UTI once in their life. Females tend to suffer more from this infection than males for several reasons: factors causing UTI among females include poor genital hygiene, small urethra, vaginal discharges, use of contraceptive devices, and unprotected intercourse.

Moges et al^11^ and Ahmad^12^ reported that the rate of MDR was very high and added that the probable contributing factors were previous catheterization, hospitalization, and antibiotics, which are also found in our study. These authors recommended a selective use of antibiotics to avoid MDR.

Regarding the causative organisms of non-MDR UTI, this is consistent with studies by Kazmi et al^10^ and Ahmad,^12^ who found that the most common pathological organisms involved in community-acquired infection included *E. coli, K. pneumoniae*, and *P. mirabilis*. To support and strengthen our finding regarding the causative organism of MDR UTI patients, after examination of 731 patients, Gajdács et al^8^ concluded that UTIs are principally caused by members of the *Enterobacterales* (*E. coli, Klebsiella spp*, and *Proteae*). Non-fermenting gram-negative bacteria are emerging as important causative agents of UTIs, primarily affecting elderly and hospitalized patients (characterized by comorbidities, catheterization), both in high- and low-income countries. The emergence of drug resistance in these pathogens should be closely monitored due to their proclivity to becoming MDR and their plasticity in drug resistance mechanisms.

Concerning antibiotic sensitivity among MDR UTI patients, Ahmad^12^ results were consistent with our study findings that antibiotic resistance was commonly observed in ampicillin (88.3%), piperacillin (72.7%), clindamycin (66.7%), amoxicillin/clavulanic acid (66.2%), and trimethoprim/sulfamethoxazole (50%).

This study found that previous UTI infection was a risk factor for MDR among the studied UTI patients. The most frequently recognized risk factors for MDR were prior antibiotic use, which was observed in 16 of 20 investigations and 25 studies with 31,284 patients with positive cultures. Previous UTIs were among the other risk variables, which is consistent with our findings.^13^

*Proteus mirabilis* (PM) infection was another risk factor for MDR in the current study’s UTI patients. In terms of importance as a cause of UTIs, PM is a common organism that results in UTIs and is ranked either fourth or fifth.^14,15^ Approximately 1-20% of all urinary pathogens are PM-UTIs in urology departments.^16^ In individuals with functional or anatomical abnormalities of the urinary tract or prolonged instrumentation, such as urinary catheterization, PM is known to lead to UTI.^17^

A previous study showed that UTIs caused by PM occurred in elderly patients, most of whom are bedridden, with long hospitalization periods and a history of recurrent UTIs.^18^ In addition, MDR-PM UTI was statistically significant in patients with episodes of prior infectious diseases.^18^

### Study limitations

Being a single center study is a limitation that could hinder the generalization of the study results.

In conclusion, based on our analyses, we conclude that UTI is more common among female patients older than 50 years old. Most of the patients had hypertension DM as a chronic disease. They were previously hospitalized and had previous UTI and catheterizati on. The most common organisms among non-MDR are *Klebsiella spp, Proteus spp*, and *E. coli*, while in the MDR patients the most common organisms are gram-negative and anaerobes. The most sensitive antibiotics are ciprofloxacin and ceftriaxone. Having UTI in ≤1 year, having an infection with PM, and being on ceftrixone treatment are risk factors for MDR. In patients with MDR UTIs and a history of UTI, PM infection or taking ceftrixone, doctors should think on administering broad-spectrum antibiotics.
